# Resonant neutron scattering lengths

**DOI:** 10.1107/S1600576724005375

**Published:** 2024-07-17

**Authors:** Robert B. Von Dreele

**Affiliations:** a Advanced Photon Source Argonne National Laboratory 9700 South Cass Avenue Lemont IL60439-4814 USA; Tohoku University, Japan

**Keywords:** resonant neutron scattering lengths, Breit–Wigner formula, rare earths

## Abstract

Unlike most of the periodic table, many rare-earth elements display considerable resonant scattering for thermal neutrons. Although this property is accompanied by strong neutron absorption, modern high-intensity neutron sources make diffraction experiments possible with these elements. Computation of scattering intensities is accomplished by fitting the variation in resonant scattering lengths (*b*_0_, *b*′ and *b*′′) to a semi-empirical Breit–Wigner formalism, which can be evaluated over the range of neutron energies useful for diffraction, typically *E* = 10–600 meV; λ = 0.4–2.8 Å (with good extrapolation to longer wavelengths).

## Abstract 2

A function and coefficients for computing resonant neutron scattering lengths for selected elements and isotopes are described.

## 1. Introduction

Most elements and common isotopes of the periodic table exhibit neutron scattering lengths that are independent of scattering vector and show no imaginary component. As they cannot be accurately calculated from first principles, but must be experimentally measured, numerous tables have been published (Koester *et al.*, 1981 ▸; Sears, 1986 ▸, 1992*a* ▸,*b* ▸; Rauch & Waschkowski, 2003 ▸). The elements/isotopes that show little or no significant neutron absorption can thus be used in the typical sample sizes (>1 cm diameter) used for diffraction with most neutron sources. However, modern high-intensity reactors and, more especially, time-of-flight (TOF) sources have allowed experiments on samples containing the few highly absorbing elements and isotopes by reducing the sample diameter to ∼1 mm (similar to X-ray sample sizes). In most cases, the high absorption arises from low-energy (<1 eV) neutron resonance features; these give rise to strong wavelength-dependent real and imaginary scattering lengths, which are not accurately provided in the usual scattering-length tables. Lynn & Seeger (1990 ▸) fitted measured values of *b*_0_ + *b*′ and *b*′′ (components of resonant neutron scattering lengths) for the rare earths, and published tables of their values over the neutron energy range of interest for diffraction (∼10–600 meV). To have them in a more convenient form, we fitted these curves to a modified Breit–Wigner function based on that of Ramaseshan (1966 ▸) and report the coefficients here. Using the same formalism, we have also included some other strongly resonant elements/isotopes.

## 2. Analysis and results

Given the resonance parameters (Table 1 ▸) from Mughabghab (1984 ▸) and Mughabghab *et al.* (1984 ▸), one can compute the real and imaginary components of the scattering length from individual resonances [equations (1)–(3)]:
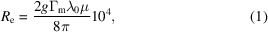

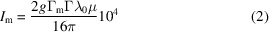
and
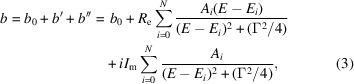
with the first term in the sums having *A*_0_ = 1.0. The term μ = (*A* + ^0^*n*)/*A*^0^*n* (*A* is atomic mass and ^0^*n* is neutron mass) is the reciprocal of the reduced mass for the nucleus–neutron interaction, and λ_0_ = (81.787/*E*_0_)^1/2^. The values of *b*_0_, *b*′, *b*′′, *R*_e_ and *I*_m_ are scaled to the conventional units for neutron scattering lengths (10^−12^ cm). Although the original Breit–Wigner formulation gave a negative imaginary component, we follow Peterson & Smith (1961 ▸) who carefully measured both X-ray and neutron resonance diffraction from a hexagonal CdS (zincite structure, space group *P*6_3_*mc*) single crystal, which made the imaginary component positive to match the chirality implied from the X-ray resonant experiment. Equation (3) was then used to fit the tabulated values of *b*_0_ + *b*′ and −*b*′′ given by Lynn & Seeger (1990 ▸) to give the coefficients shown in Table 2 ▸; in the fit *A*_0_ = *B*_0_ = 1.0. The residuals for these fits were all less than 1%. Lynn & Seeger (1990 ▸) followed the original Breit–Wigner negative imaginary sign convention; the sign only matters for single-crystal diffraction experiments on chiral materials where Friedel’s law does not hold (*e.g.* hexagonal CdS, as noted above).

## 3. Discussion

**Figure 1:**
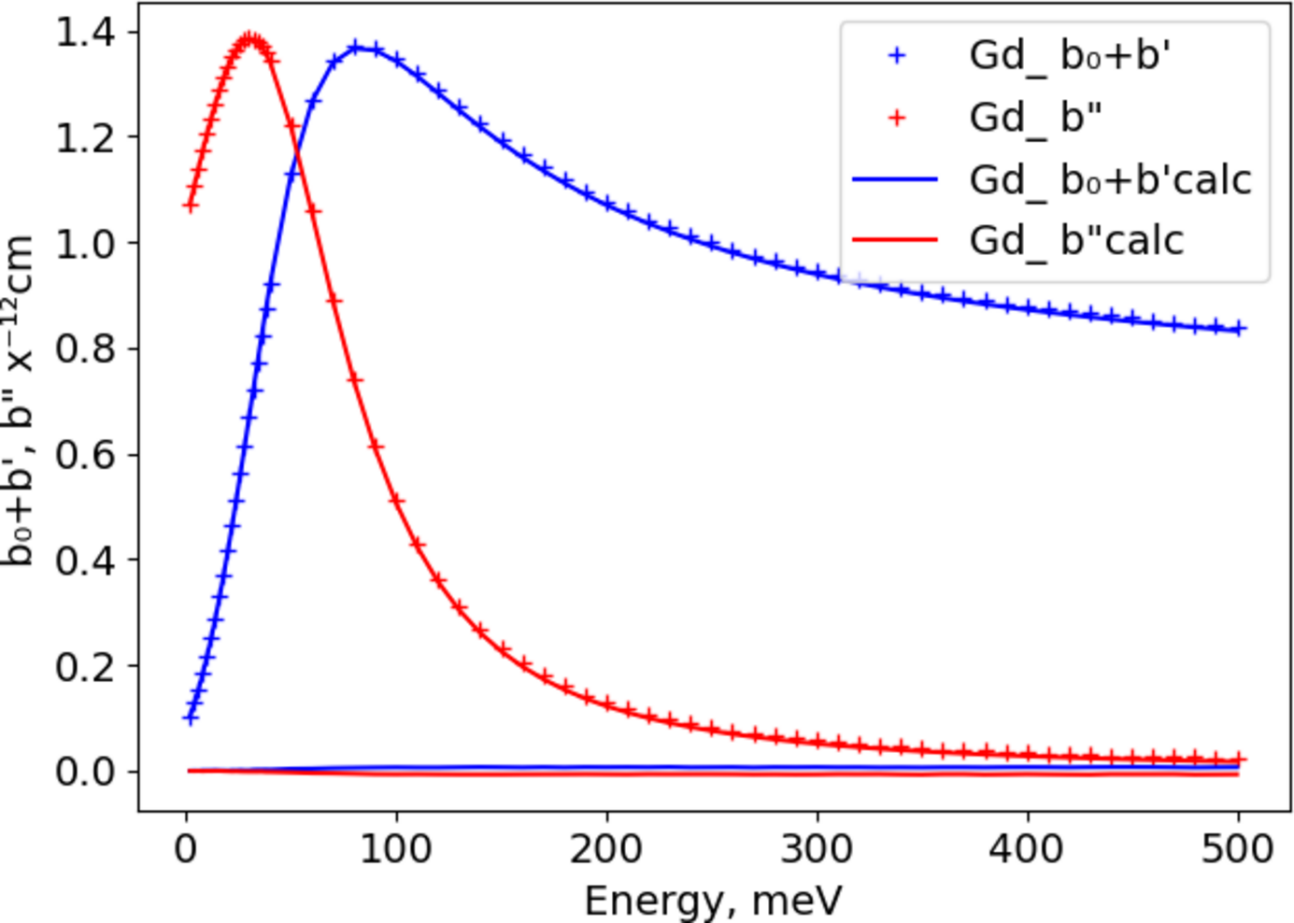
Fits of equation (3) to Lynn & Seeger (1990 ▸) resonant neutron scattering lengths for naturally abundant Gd with respect to energy; the real part (*b*_0_ + *b*′) is in blue and the imaginary part (*b*′′) is in red. Crosses mark values from Lynn & Seeger (1990 ▸) with the sign of *b*′′ reversed; curves are from the fitted coefficients. The respective residuals from the fits are shown as blue and red lines close to zero.

Apart from Eu and Er, a single resonance term was sufficient to form a good fit between equation (3) and the Lynn & Seeger (1990 ▸) resonant scattering curves. For example, for Gd (see Fig. 1 ▸), which has two resonant isotopes (^155^Gd and ^157^Gd), the best-fit values of *R*_e_ and *I*_m_ (72.72 and 3866, respectively) are close to the sum of isotope-abundance-weighted values of *R*_e_ and *I*_m_ from Table 1 ▸ (73.1 and 3708, respectively). Both the resonance energies and widths are similar for these isotopes (Table 1 ▸), and thus the values obtained (Table 2 ▸) for naturally abundant Gd are their weighted averages. The best-fit value of *b*_0_ for Gd has abundance-weighted contributions from all seven naturally occurring Gd isotopes. The *R*_e_, *I*_m_, *E*_0_ and Γ parameters (Table 2 ▸) for the fits to the ^155^Gd and ^157^Gd curves are in good agreement with the values in Table 1 ▸. Similar agreement is found for the other elements/isotopes with a single resonance. This includes Sm, even though there is a second resonance at 872 meV; it is sufficiently higher than the practical thermal region (<500 meV; >0.4 Å) explored here so it has little or no impact on the resonant scattering lengths.

**Figure 2:**
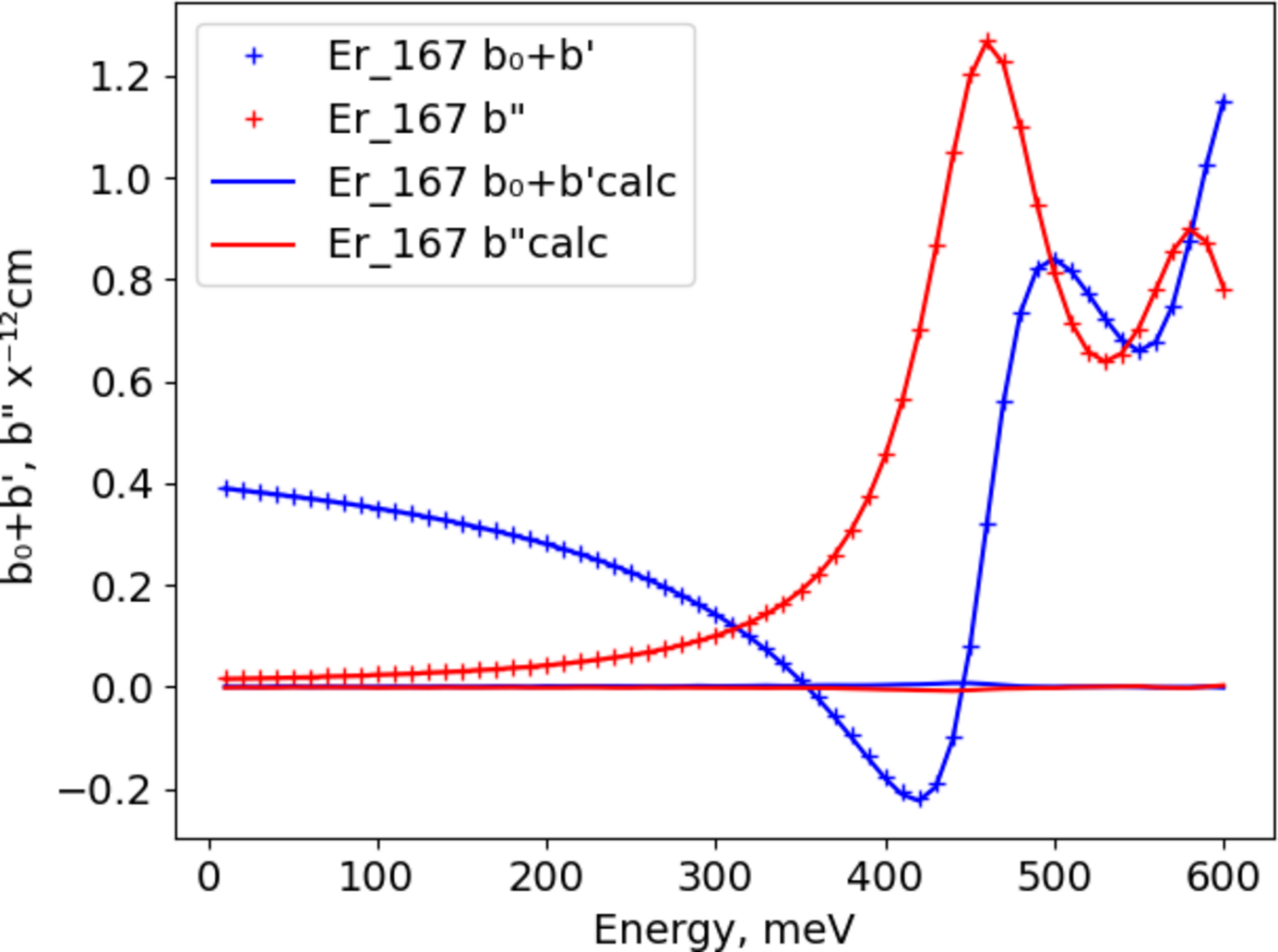
Fits of equation (3) to Lynn & Seeger (1990 ▸) resonant neutron scattering lengths for ^167^Er with respect to energy; the real part (*b*_0_ + *b*′ ) is in blue and the imaginary part (*b*′′) is in red. Crosses mark values from Lynn & Seeger (1990 ▸) with the sign of *b*′′ reversed; curves are from the fitted coefficients. The respective residuals from the fits are shown as blue and red lines close to zero.

For ^167^Er and Eu, each has two resonances within the thermal neutron energy band; this requires the use of two terms in equation (3). An example fit for ^167^Er is shown in Fig. 2 ▸ using the parameters *b*_0_, *R*_e_, *I*_m_, *E*_0_, Γ, *E*_1_ and *A*_1_ (Table 2 ▸); the first six correspond well to the parameters given in Table 1 ▸. *A*_1_ is simply the ratio of the *R*_e_ values for the *E*_1_ resonance and the *E*_0_ resonance; values from Table 1 ▸ give 0.6209 while the best-fit value given in Table 2 ▸ is 0.6481 (11). We have constrained the widths (Γ) of the two resonances to be the same; they are nearly identical for ^167^Er (Table 1 ▸), so this is a suitable simplifying assumption. The same set of coefficients is used for naturally abundant Er; its best-fit values (Table 2 ▸) are similar to the isotopic values, except that *R*_e_ and *I*_m_ are scaled by the natural abundance of ^167^Er (22.9%).

**Figure 3:**
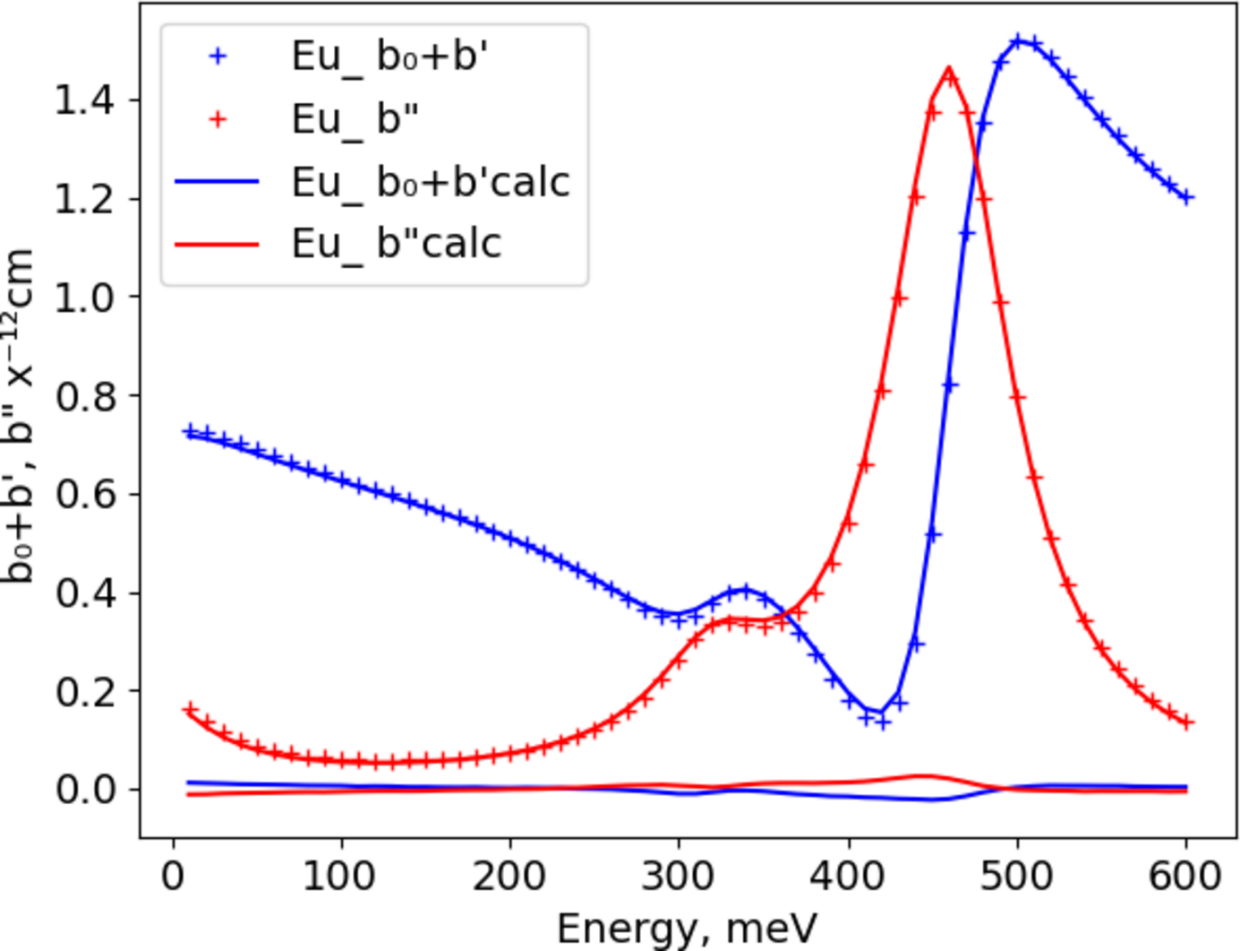
Fits of equation (3) to Lynn & Seeger (1990 ▸) resonant neutron scattering lengths for naturally abundant Eu with respect to energy; the real part (*b*_0_ + *b*′) is in blue and the imaginary part (*b*′′) is in red. Crosses mark values from Lynn & Seeger (1990 ▸) with the sign of *b*′′ reversed; curves are from the fitted coefficients. The respective residuals from the fits are shown as blue and red lines close to zero.

For Eu, the rise in *b*′′ at low energy (Fig. 3 ▸) indicates the existence of a possible ‘zero’ energy bound state for the ^151^Eu + ^0^*n* collision (Lynn, 1989 ▸), which is evident in the work of Mughabghab (1984 ▸) as a small negative *E*_0_ term in the list of resonances. For the present work, naturally abundant Eu and ^151^Eu require an additional third term in equation (3) for this zero-bound state. The apparent best-fit value of *E*_2_ = −31 meV (Table 2 ▸) is close to that reported by Lynn (1989 ▸) (−34 meV) from a multilevel analysis. We have assumed that all three terms have the same resonance width (Γ). Thus the best-fit value is close to that of the dominant *E* = 460 meV resonance. The best-fit values of *R*_e_ and *I*_m_ are reasonable compared with the corresponding ones for the *E* = 321 meV resonance in Table 1 ▸. The corresponding values of *R*_e_ and *I*_m_ for the dominant *E* = 460 meV resonance, as given by the best-fit *R*_e_*A*_1_ and *I*_m_*A*_1_ (from Table 2 ▸), respectively, are quite close to those in Table 1 ▸. There is good agreement between the best-fit values for naturally abundant Eu and ^151^Eu apart from *R*_e_ and *I*_m_, which scale by the natural abundance (47.89%) of ^151^Eu. The higher-energy resonance (*E* = 1055 meV) in ^151^Eu has no impact on the thermal neutron scattering lengths.

In all of these fits, the agreement between the simplified model used here and the values given by Lynn & Seeger (1990 ▸) is best for elements and isotopes with a single resonance (*cf*. Fig. 1 ▸); the largest differences (|Δ*b*| < 0.01 × 10^−12^ cm) are at the resonance. For two or more resonances, the differences can be larger (|Δ*b*| < 0.05 × 10^−12^ cm; compare Figs. 2 ▸ and 3 ▸) but are generally confined to be near the resonances.

Given the effectiveness of the Breit–Wigner expression as used in equation (3) for describing resonant neutron scattering lengths, this function and its coefficients have been implemented in the software tools *GSAS* (Larson & Von Dreele, 2004 ▸; Toby, 2001 ▸) and *GSAS-II* (Toby & Von Dreele, 2013 ▸). Also included in this present study are coefficients (Table 1 ▸) for the non-rare earths Rh, Cd and Pu, which also have low-energy resonances and thus wavelength-dependent scattering lengths.

## 4. Conclusions

Since the original implementation of this function and its coefficients in *GSAS* (Larson & Von Dreele, 2004 ▸) and their subsequent inclusion in *GSAS-II*, there have been a few studies showing their use. One is a study of ErD_2_ thin films by Rodriguez *et al.* (2006 ▸), which used *GSAS* to analyse neutron TOF diffraction data. Over the wavelength range of this experiment (0.48 < λ < 4.8 Å), the resonant scattering lengths of Er were −0.129 < *b*′ < −0.040 and −0.74 < *b*′′ < −0.172 (*b*_0_ = 0.850 × 10^−12^ cm). Magnetic structures of Sm_3_Ag_4_Sn_4_ and Gd_3_Ag_4_Sn_4_ were determined by Ryan & Cranswick (2008 ▸) using *GSAS*. Since their interest was in magnetic structures, they chose a long wavelength (2.37 Å) and a thin flat-plate geometry to reduce the impact of absorption; correct resonant scattering lengths are essential for these magnetic structure determinations. More recent is a study of ErHO by Zapp *et al.* (2021 ▸) using *GSAS-II* with constant wavelength (λ = 1.155 Å) neutron powder data. They noted that the coherent scattering length of Er at this wavelength was ∼4% higher than the value given by Sears (1992*a* ▸) for λ = 1.798 Å.

From these examples, it is evident that neutron powder diffraction experiments involving these highly absorbing and strongly resonant elements and isotopes are feasible and the function and coefficients described in this work are useful. However, to be successful, one must have good knowledge of the neutron wavelength. This presents no problem for constant-wavelength experiments but can be problematic for neutron TOF, where the wavelength must be inferred from the experimental TOF via the de Broglie relation and the total neutron flight path. Most TOF powder diffractometers arrange the detectors into ‘banks’ that cover a particular solid angle with a particular range in the scattering angle, 2Θ. The neutron events from the individual detector pixels in a bank are collected onto a common TOF scale, compensating for the differences in 2Θ by applying shifts in TOF (a process known as ‘electronic time focusing’) so that Bragg peaks observed in all detector pixels all fall at the same TOF in the combined dataset reported at a nominal 2Θ assigned to that bank. This combines the scattering of neutrons that have different wavelengths. The wider the bank in scattering angle, the greater these TOF shifts will be and the greater the span in wavelength. The wavelength spread for a given *d* spacing will depend on the angular range, ΔΘ, encompassed by the detector bank and its nominal scattering angle, 2Θ:
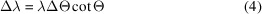
or, by substituting Bragg’s law for λ,
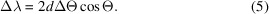
These effects are negligible at high scattering angles but can be quite significant at low angles and with detector banks that cover a wide scattering angle; therefore, the inferred values of *b*′ and *b*′′ using the results of this work may be significantly compromised. Similarly, this process of combining observations made at differing 2Θ values also interferes with the treatment of other wavelength- and angle-dependent phenomena, such as absorption and extinction. It may be better for TOF instruments with wide-angle detector banks to do electronic angle focusing to a narrow wavelength band, thus creating a pseudo-constant-wavelength powder diffraction pattern with scattering angle as the independent variable.

## Figures and Tables

**Table 1:** Low-energy neutron resonance parameters (*E*_0_, 2*g*Γ_m_ and Γ) for selected isotopes taken from Mughabghab (1984 ▸) and Mughabghab *et al.* (1984 ▸), and *R*_e_ and *I*_m_ computed according to equations (1) and (2)

Isotope	Natural abundance (%)	μ	*E*_0_ (meV)	2*g*Γ_m_	Γ (meV)	*R*_e_	*I*_m_
^103^Rh	100	1.0097	1257.0	0.77	156	78.92	6156.0
^113^Cd	12.22	1.0088	178.0	0.98	113	226.7	15070.0
^149^Sm	13.9	1.0067	97.3	0.600	60.5	220.4	6667.0
^149^Sm	13.9	1.0067	872	0.835	59.8	102.3	3060
^151^Eu	47.86	1.066	321	0.0833	74.5	16.84	627.4
^151^Eu	47.86	1.066	460	0.776	87.0	131.1	5702.0
^151^Eu	47.86	1.066	1055	0.2254	88.0	25.14	1106.0
^155^Gd	14.8	1.0065	26.8	0.130	108.0	90.96	4912.0
^157^Gd	15.7	1.0065	31.4	0.588	106.0	380.0	20143.0
^167^Er	22.9	1.0060	460	0.314	88.0	53.00	2332.0
^167^Er	22.9	1.0060	584	0.224	86.3	33.56	1448.0
^168^Yb	0.127	1.0060	597	4.4	64.0	652.0	20863.0
^176^Lu	2.9	1.0057	1413	0.0865	62.3	26.34	820.4
^239^Pu	N/A	1.0042	296	0.108	102.0	22.66	1156.0
^240^Pu	N/A	1.0041	1057	4.64	32.4	515.8	8356.0

**Table 2:** Low-energy neutron resonance coefficients for rare-earth elements and isotopes from fitting equation (3) to the resonance tables of Lynn & Seeger (1990 ▸)

Element/isotope	*b*_0_	*R*_e_	*I*_m_	*E*_0_ (meV)	Γ (meV)	*A*_1_	*E*_1_ (meV)	*A*_2_	*E*_2_ (meV)
Sm	0.4099 (10)	30.10 (15)	949 (6)	97.75 (13)	65.14 (23)	0	0	0	0
^149^Sm	0.470 (8)	216.4 (11)	6830 (50)	97.74 (13)	65.15 (23)	0	0	0	0
Eu	0.7550 (23)	8.55 (4)	385.5 (33)	321.01 (21)	87.32 (11)	7.14 (5)	459.65 (7)	1.25 (7)	−31.0 (8)
^151^Eu	0.731 (4)	17.88 (9)	807 (6)	321.0 (20)	87.35 (11)	7.14 (4)	459.64 (6)	1.22 (6)	−30.5 (8)
Gd	0.6794 (11)	72.72 (31)	3866 (13)	30.40 (7)	105.60 (23)	0	0	0	0
^155^Gd	0.6820 (2)	88.74 (5)	4672.3 (19)	28.069 (8)	105.135 (27)	0	0	0	0
^157^Gd	0.6428 (18)	379.9 (5)	20178 (18)	31.0194 (20)	105.74 (6)	0	0	0	0
Er	0.8525 (2)	12.08 (3)	523.5 (13)	460.30 (7)	88.08 (9)	0.6522 (21)	584.42 (8)	0	0
^167^Er	0.5635 (5)	52.42 (6)	2281.9 (28)	460.16 (4)	87.93 (5)	0.6481 (11)	584.23 (4)	0	0
Yb	1.2273 (8)	0.56 (10)	36.2 (33)	596.97†	66.165†	0	0	0	0
^168^Yb	0.7046 (1)	635.179 (12)	21152.9 (7)	596.9696 (5)	66.1647 (12)	0	0	0	0
^176^Lu	0.8042 (13)	25.55 (16)	765 (8)	141.36 (18)	60.560 (31)	0	0	0	0

† Values for Yb taken from ^168^Yb, since the resonant isotope abundance is only 0.127%.
